# Supplementary material for: Heterozygous expression of a Kcnt1 gain-of-function variant has differential effects on SST- and PV-expressing cortical GABAergic neurons
Source: bioRxiv. 2023 Oct 11:2023.10.11.561953. Preprint. [Version 1] doi: 10.1101/2023.10.11.561953 (PMC10592778; doi:10.1101/2023.10.11.561953)
Supplement: Supplement 1 [file NIHPP2023.10.11.561953v1-supplement-1.pdf]

bioRxiv preprint doi: https://doi.org/10.1101/2023.10.11.561953; this version posted October 11, 2023. The copyright holder for this preprint (which was not certified by peer review) is the author/funder, who has granted bioRxiv a license to display the preprint in perpetuity. It is made available under aCC-BY 4.0 International license.

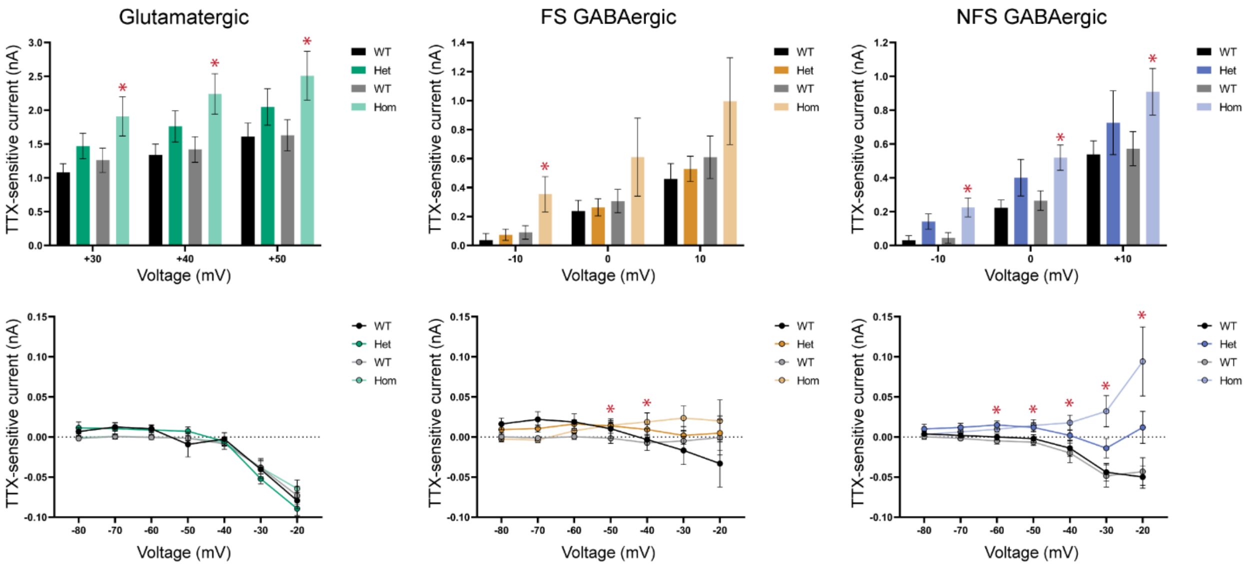

Supplemental Figure 2-1. The magnitudes of the K_{Na} current increases in YH-HET glutamatergic and NFS GABAergic neurons are intermediate to those of WT and YH-HOM neurons. Bar graphs (top three) illustrate differences in K_{Na} current (mean ± SEM) between YH-HET [dark color; WT littermates (black)] and YH-HOM [light color; WT littermates (gray)] neurons for voltage steps where significant increases were previously observed (red asterisks where $p < 0.05$) in glutamatergic, and FS and NFS GABAergic, YH-HOM neurons. Line graphs (bottom three) illustrate differences in K_{Na} current (mean ± SEM) between YH-HET [dark color; WT littermates (black)] and YH-HOM [light color; WT littermates (gray)] neurons across negative voltage steps (-80 to -20 mV; red asterisks where $p < 0.05$ in YH-HOM compared with WT) in glutamatergic, and FS and NFS GABAergic, neurons.

bioRxiv preprint doi: https://doi.org/10.1101/2023.10.11.561953; this version posted October 11, 2023. The copyright holder for this preprint (which was not certified by peer review) is the author/funder, who has granted bioRxiv a license to display the preprint in perpetuity. It is made available under aCC-BY 4.0 International license.

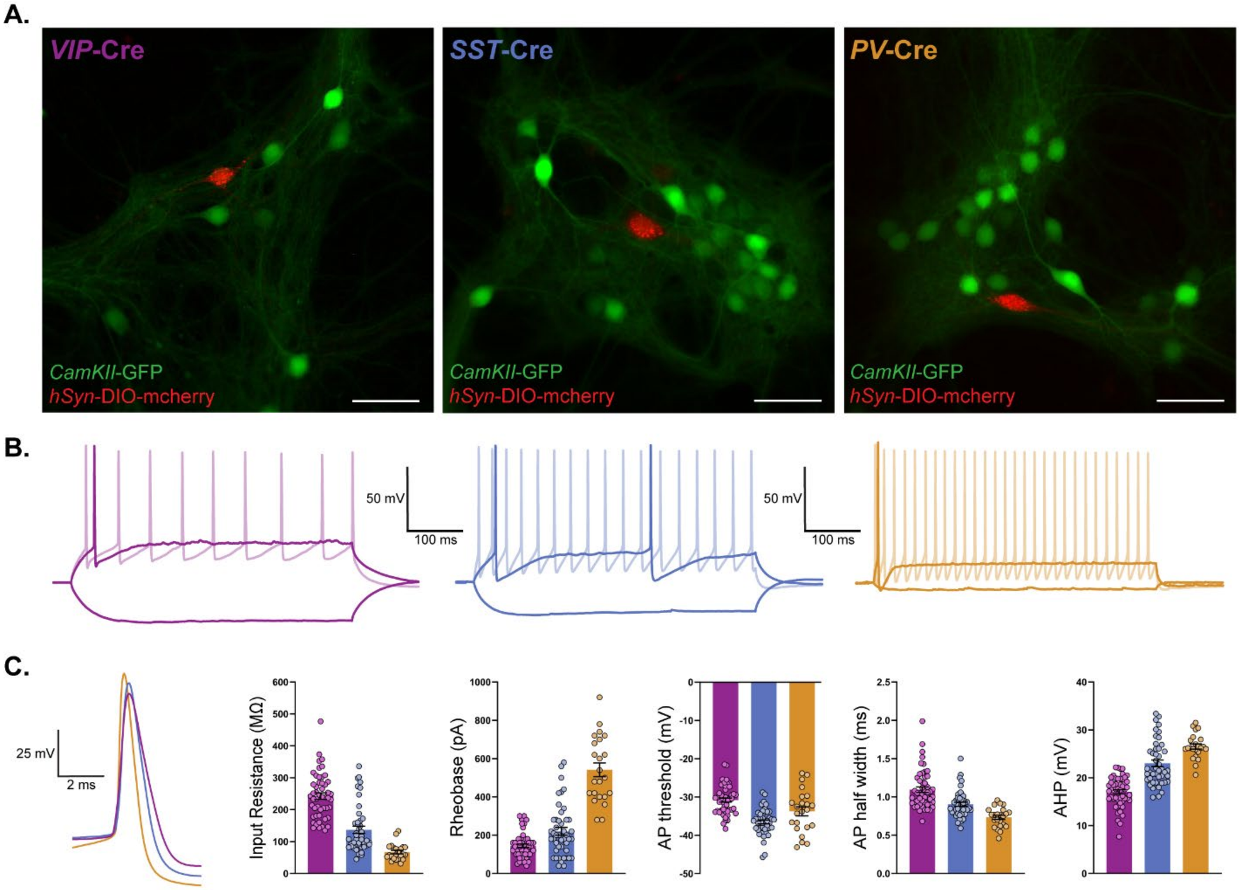

Supplemental Figure 4-1. Cre recombinase-expressing, cortical GABAergic neuron subpopulations retain characteristic electrophysiological features in culture. Cortical neurons isolated and cultured from P0 pups (*Kcnt1*$^{+/+}$) from VIP-, SST-, or PV-Cre mouse lines were infected with AAV-*CamKII*-GFP to label glutamatergic neurons, and AAV-*hSyn*-DIO-mCherry to label Cre-expressing neurons, at DIV 1. **(A)** Images show GFP^{+} glutamatergic neurons and mCherry^{+} VIP-, SST-, or PV-expressing neurons (left to right) at DIV 14 (VIP and SST) or DIV 16 (PV). The scale bars represent 50 µm. **(B)** Representative responses to step currents are shown for VIP (purple), SST (blue), and PV (orange) neurons (left to right). For each neuron type, the superimposed dark traces illustrate the input resistance (in response to a depolarizing step) and the rheobase (the first trace with an AP in response to a hyperpolarizing step), and the light trace shows the first step current response to induce repetitive AP firing across the step. **(C)** On the left, the first AP of each rheobase trace is shown for VIP (purple), SST (blue), and PV (orange) neurons. On the right, bar graphs show quantification of the membrane properties and AP parameters for each neuron type (VIP, SST, and PV, left to right), with individual neuron measurements overlaid in scatter plots.

bioRxiv preprint doi: https://doi.org/10.1101/2023.10.11.561953; this version posted October 11, 2023. The copyright holder for this preprint (which was not certified by peer review) is the author/funder, who has granted bioRxiv a license to display the preprint in perpetuity. It is made available under aCC-BY 4.0 International license.

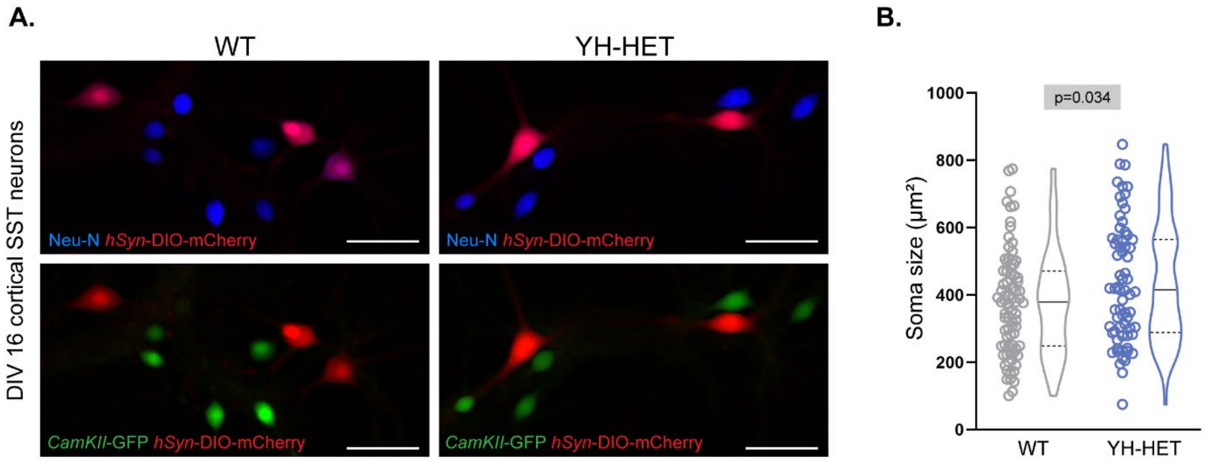

Supplemental Figure 4-2. YH-HET SST neurons have increased soma size compared with those of WT. Cortical neurons were isolated and cultured from P0 pup progeny (WT and YH-HET) of YH-HET mice crossed to SST-Cre mice. Neurons were infected with AAV-*CamKII*-GFP to label glutamatergic neurons, and AAV-*hSyn*-DIO-mCherry to label Cre-expressing neurons, at DIV 1, and immunostained using anti-Neu-N antibodies at DIV 16. **(A)** Top two images show mCherry^{+} SST-expressing neurons as a subpopulation of Neu-N^{+} cortical neurons, and bottom two images show GFP^{-}, mCherry^{+} SST-expressing neurons intermingled with GFP^{+} glutamatergic neurons. The scale bars represent 50 μm. **(B)** A graph shows individual neuron measurements and summary violin plots of the mCherry^{+} neuron soma size (WT, gray; YH-HET, blue) with the p-value displayed at the top.

bioRxiv preprint doi: https://doi.org/10.1101/2023.10.11.561953; this version posted October 11, 2023. The copyright holder for this preprint (which was not certified by peer review) is the author/funder, who has granted bioRxiv a license to display the preprint in perpetuity. It is made available under aCC-BY 4.0 International license.

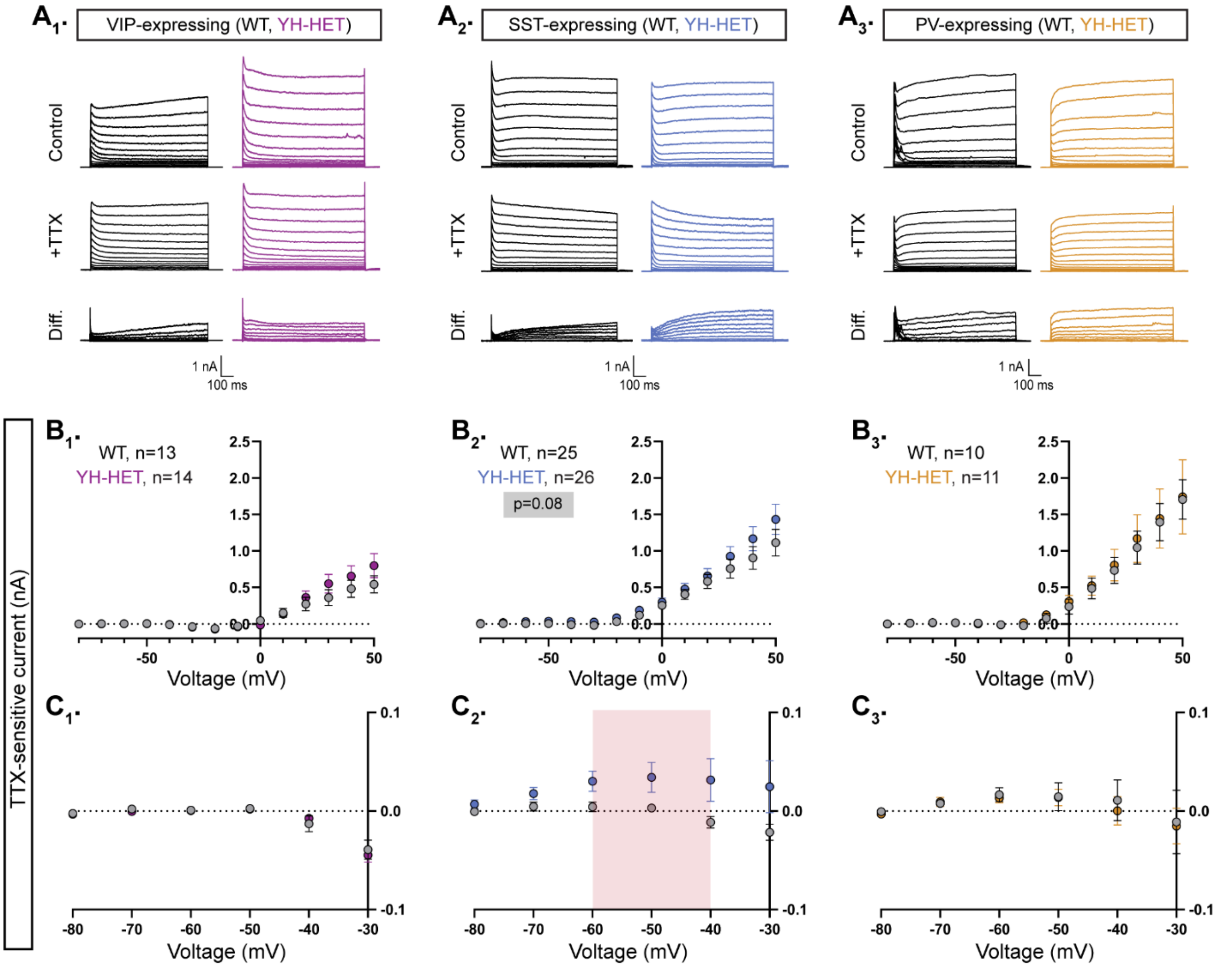

Supplemental Figure 5. The *Kcnt1*-Y777H variant increases K_{Na} currents measured with TTX subtraction across subthreshold voltages in SST-expressing GABAergic neurons. (A_1-A_3) Representative traces in control (top), 0.5 µM TTX (middle), and the difference current (bottom) calculated by subtracting the membrane current response to voltage steps (-80 to +50 mV) from a holding potential of -70 mV in TTX from the response in control external solution in VIP-, SST-, and PV-expressing, WT (black) and YH-HET (colors) neurons. **(B_1-B_3)** Summary data shows the K_{Na} current (mean ± SEM) for each voltage step in VIP-, SST-, and PV-expressing, WT (black and gray) and YH-HET (colors) neurons. The p-values are shown on each graph where $p < 0.05$, and the n values are the number of neurons recorded for each group. **(C_1-C_3)** Plots of the K_{Na} current (mean ± SEM) for each voltage step from -80 to 0 mV in WT (black and gray) and YH-HET (colors) neurons to illustrate the values that are too small to be seen on the graphs in B_1-B_3. The shaded red area in C_2 indicates the subthreshold voltage range with significantly higher K_{Na} currents in YH-HET relative to WT neurons. Statistical significance for I-V plots was tested using Generalized Linear Mixed Models with genotype and current step as fixed effects followed by pairwise comparisons at each level.

bioRxiv preprint doi: https://doi.org/10.1101/2023.10.11.561953; this version posted October 11, 2023. The copyright holder for this preprint (which was not certified by peer review) is the author/funder, who has granted bioRxiv a license to display the preprint in perpetuity. It is made available under aCC-BY 4.0 International license.

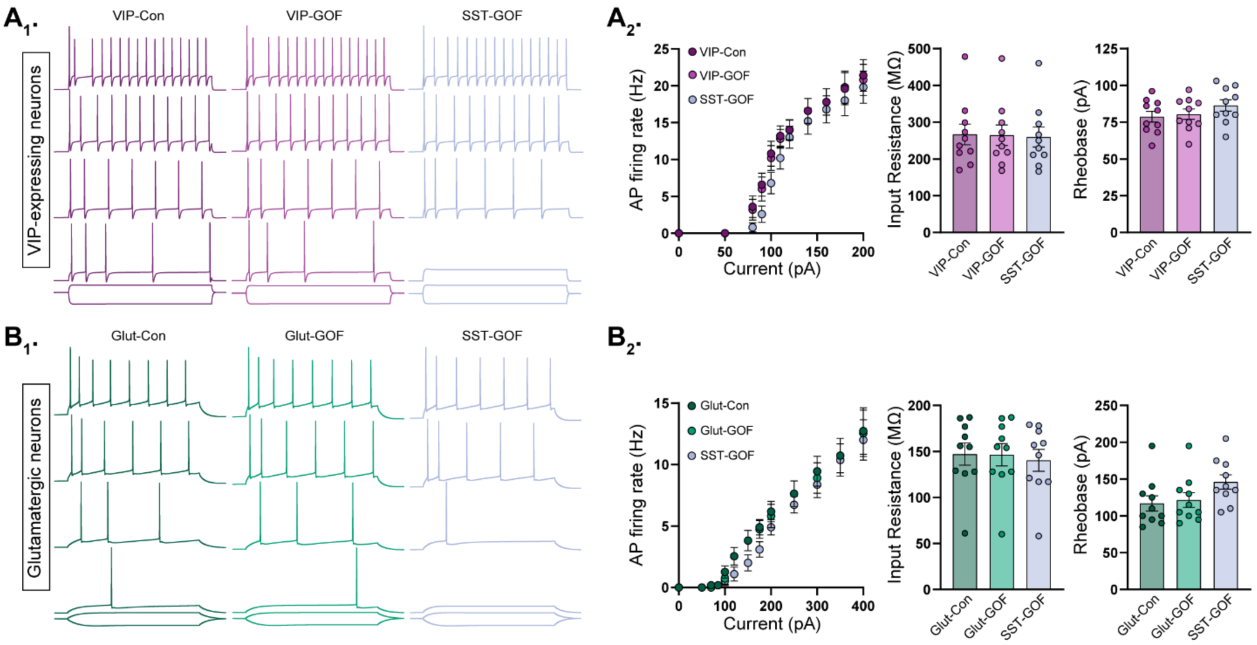

Supplemental Figure 6. Model VIP and glutamatergic neurons with insertion of SST-like, KCNT1 GOF kinetics show reduced excitability. (A_1) Simulated traces from model VIP neurons, using VIP KCNT1 activation curve parameters (purple) or SST KCNT activation curve parameters (blue), in response to 500-ms current injections at two levels of Na^+-sensitivity (Con-40 mM and GOF-30mM). Representative traces are shown at increasing current steps from bottom to top for each level. **(A_2)** Summary data from 10 model VIP neurons, with VIP KCNT1 parameters (VIP-Con and VIP-GOF) or SST KCNT1 parameters (SST-GOF) showing (from left to right) the number of APs at different current steps (F-I plot), the input resistance, and rheobase. The bar graphs (mean ± SEM) are overlaid with scatter plots of individual neuron measurements. **(B_1)** Simulated traces from model glutamatergic neurons, using glutamatergic KCNT1 activation curve parameters (green) or SST KCNT activation curve parameters (blue), in response to 500-ms current injections at two levels of Na^+-sensitivity (Con-40 mM and GOF-30mM). Representative traces are shown at increasing current steps from bottom to top for each level. **(B_2)** Summary data from 10 model glutamatergic neurons, with glutamatergic KCNT1 parameters (Glut-Con and Glut-GOF) or SST KCNT1 parameters (SST-GOF) showing (from left to right) the number of APs at different current steps (F-I plot), the input resistance, and rheobase. The bar graphs (mean ± SEM) are overlaid with scatter plots of individual neuron measurements.

bioRxiv preprint doi: https://doi.org/10.1101/2023.10.11.561953; this version posted October 11, 2023. The copyright holder for this preprint (which was not certified by peer review) is the author/funder, who has granted bioRxiv a license to display the preprint in perpetuity. It is made available under aCC-BY 4.0 International license.

Table S1. Electrophysiological parameters of current clamp recordings from neuronal cultures.

	Glutamatergic			FS GABAergic			NFS GABAergic		
	WT	YH-HET		WT	YH-HET		WT	YH-HET	
	N = 9	N = 9		N = 9	N = 9		N = 9	N = 9	
	n = 27	n = 30	p-value	n = 27	n = 29	p-value	n = 26	n = 22	p-value
V_{rest} (mV)	-57.9±1.6	-57.0±1.7	0.61	-53.8±1.8	-52.8±1.8	0.60	-56.3±1.8	-54.4±1.9	0.34
R_{in} (MΩ)	177±17	182±16	0.80	95±9	105±10	0.40	171±16	146±14	0.19
Tau (ms)	32.9±3.8	39.3±4.3	0.14	17.3±2.0	16.7±1.9	0.75	24.9±2.9	22.2±2.7	0.37
C_m (pF)	198±15	215±16	0.23	183±14	163±12	0.15	143±11	164±13	0.09
Rheo (pA)	171±19	148±16	0.25	400±45	415±47	0.78	196±22	297±36	**0.005**
AP_{thresh} (mV)	-28.2±1.5	28.3±1.5	0.93	-25.7±1.6	-25.8±1.6	0.95	-28.2±1.6	-24.4±1.7	**0.012**
AP_{amp} (mV)	77.8±2.0	77.5±1.5	0.51	70.9±1.8	67.8±1.7	0.15	71.5±1.8	68.7±1.9	0.28
AP_{hw} (ms)	2.19±0.12	2.20±0.12	0.95	0.89±0.05	0.91±0.05	0.85	1.36±0.08	1.11±0.07	**0.01**
AHP (mV)	11.8±0.6	11.8±0.6	0.96	27.5±1.3	26.9±1.4	0.78	19.3±1.0	22.6±1.2	**0.034**
AP_{mfr} (Hz)	22.4±2.3	22.4±2.4	0.99	68.8±6.2	71.4±6.6	0.53	35.0±3.8	31.8±3.5	0.25

For an explanation of the parameters, see Methods. R_{in} = input resistance, Tau = membrane time constant, Rheo = rheobase current, C_m = membrane capacitance, AP = action potential, thresh = threshold, amp = amplitude, hw = half width, AHP = afterhyperpolarization, and mfr = maximum firing rate. Values shown are estimated marginal means ± the standard error as determined by implementing a Generalized Linear Mixed Model. P values less than 0.05 are in red, bold type. For each subgroup, N values are the number of mouse pups, and n values are the number of neurons.

Table S2. Electrophysiological parameters of current clamp recordings from neuronal cultures of three major GABAergic subtypes.

	SST^+ GABAergic			VIP^+ GABAergic			PV^+ GABAergic		
	WT	YH-HET		WT	YH-HET		WT	YH-HET	
	N = 4	N = 5		N = 3	N = 4		N = 3	N = 4	
	n = 45	n = 46	p-value	n = 50	n = 48	p-value	n = 23	n = 25	p-value
R_{in} (MΩ)	133±18	95±13	**0.001**	243±10	249±11	0.645	67±4	66±3	0.850
Tau (ms)	16.9±1.1	13.5±0.8	**0.012**	35.1±2.4	33.1±2.3	0.384	6.7±0.5	6.0±0.4	0.302
C_m (pF)	135±13	152±15	**0.032**	148±6	134±6	0.055	103±5	90±4	0.050
Rheo (pA)	232±41	319±56	**0.001**	149±12	157±13	0.344	546±33	441±24	**0.031**
AP_{thresh} (mV)	-36.4±0.8	-35.2±0.8	0.113	-30.9±0.4	-31.0±0.4	0.850	-34.2±1.8	-35.5±1.9	0.308
AP_{amp} (mV)	79.9±1.5	80.2±1.5	0.850	75.6±0.9	75.5±0.9	0.943	77.6±1.0	82.7±1.0	**0.002**
AP_{hw} (ms)	0.91±0.02	0.86±0.02	0.142	1.09±0.04	1.09±0.04	0.916	0.73±0.03	0.69±0.02	0.180
AHP (mV)	23.0±0.7	23.5±0.7	0.554	17.2±0.9	17.4±0.9	0.860	26.6±0.6	25.3±0.5	0.150
AP_{mfr} (Hz)	57.6±5.1	55.7±4.9	0.432	35.5±2.8	35.9±2.8	0.857	69.4±4.9	79.0±5.3	0.068

For an explanation of the parameters, see Methods. R_{in} = input resistance, Tau = membrane time constant, Rheo = rheobase current, C_m = membrane capacitance, AP = action potential, thresh = threshold, amp = amplitude, hw = half width, AHP = afterhyperpolarization, and mfr = maximum firing rate. Values shown are estimated marginal means ± the standard error as determined by implementing a Generalized Linear Mixed Model. P values less than 0.05 are in red, bold type. For each subgroup, N values are the number of mouse pups, and n values are the number of neurons.
